# Effect of Chemical Treatment and Length of Raffia Fiber (*Raphia vinifera*) on Mechanical Stiffening of Polyester Composites

**DOI:** 10.3390/polym12122899

**Published:** 2020-12-03

**Authors:** Edwillson Gonçalves de Oliveira Filho, Fernanda Santos da Luz, Roberto Tetsuo Fujiyama, Alisson Clay Rios da Silva, Verônica Scarpini Candido, Sergio Neves Monteiro

**Affiliations:** 1 Engineering of Natural Resources of the Amazon Program, Federal University of Pará—UFPA, Rua Augusto Corrêa, 01, Belém, Pará 66075-110, Brazil; edwillson.goncalves@gmail.com.br (E.G.d.O.F.); fujiyama.ufpa@gmail.com (R.T.F.); alissonrios@ufpa.br (A.C.R.d.S.); scarpini@ufpa.br (V.S.C.); 2 Materials Science Program, Military Institute of Engineering—IME, Praça General Tibúrcio 80, Urca, Rio de Janeiro 22290-270, Brazil; snevesmonteiro@gmail.com

**Keywords:** natural fiber composite, raffia fiber, *Raphia vinifera*, mechanical properties, stiffening effect

## Abstract

In recent decades, the unique characteristics of natural fibers have promoted their use as reinforcement in polymeric composites. This is verified in several industrial sectors, from packaging to automotive and civil construction. Among the natural fibers, the raffia fiber extracted from the palm tree *Raphia vinifera* and introduced in the Amazon region a long time ago; started to be considered for the production of polymeric composites only in recent years. For the first time, the effect of raffia fiber length and its alkali treatment on the mechanical properties of a polymer composite was disclosed. Tensile tests were performed in composites with raffia fibers randomly dispersed into terephthalate-based unsaturated polyester resin. The results showed an increase in the Young’s moduli, confirmed by ANOVA, for the composite with both untreated and alkali-treated fibers in comparison to the plain polyester, which characterizes a stiffening effect. The composites with alkali treated fibers exhibited similar tensile strength values for all lengths; however, their strengths are lower than those for the untreated condition due to a weak raffia fiber/polyester matrix adhesion. Therefore, this work fills the current knowledge gap on raffia fiber incorporation in polyester matrix and valorizes this abundant Brazilian resource, providing additional information towards the use of raffia fiber in polymer composites.

## 1. Introduction

The use of renewable and biodegradable materials has advanced remarkably in recent years. Among these, the natural lignocellulosic fibers (NLFs) have stood out as a sustainable alternative to replace synthetic fiber in polymeric composites [1,2,3,4,5,6,7,8,9,10,11,12] in the most diverse areas of civil construction [13], the automotive industry [14,15,16], and ballistic vests [17,18,19,20]. Many NLFs have been traditionally used by local people in developing regions as craftwork, ropes, or considered to be industrial waste, such as sugarcane bagasse fiber [21], coir fiber [22,23] and PALF [23]. The growing interest in NLFs is due to their characteristics of relatively low cost, low density, flexibility, and non-abrasive behavior, which unlike synthetic fibers, avoid damage to the processing equipment. In addition, NLFs help with socio-environmental issues, as they come from renewable resources, are biodegradable, and are a source of income in developing regions [24,25].

**Figure 1:**
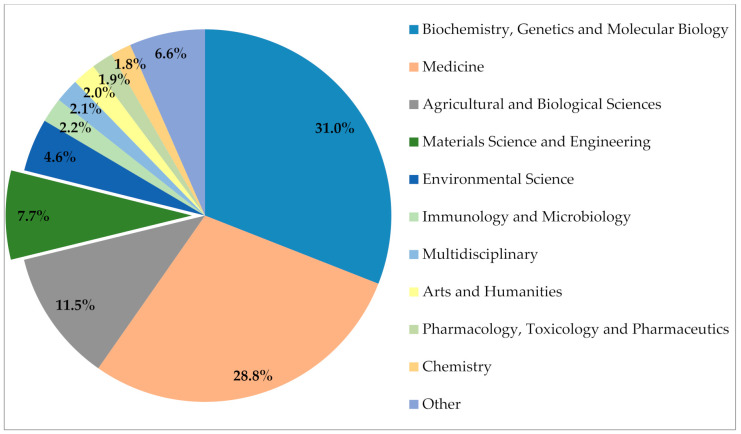
Number of publications with keywords “raffia” or “raphia” by subject area reported by the Scopus database between 1910 and 2020 [26].

Although several studies on the use of NLFs as reinforcement in composites have been reviewed [1,2,3,4,5,6,7,8,9,10,11,12], relatively few papers on the potential use of raffia palm fibers in polymer composites were published so far. In fact, according to the Scopus database [26], in 2020 only 100 documents appear with the keywords “raffia” or “raphia” in the engineering and materials science subject areas [27,28,29,30]. This represents less than 8% of all publications on raffia, as shown in Figure 1.

**Figure 2:**
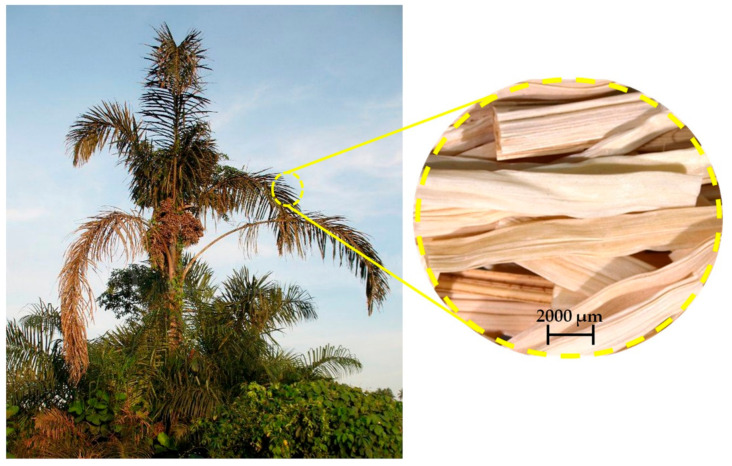
*Raphia vinifera* palm tree [44] and raffia fiber extracted from the leaf (detail).

The raffia fiber extracted from the leaf palm tree, illustrated in Figure 2, is a native from the African continent with over 20 recognized species. Among these species, the *Raphia vinifera*, was introduced centuries ago in the north of Brazil. This palm tree reaches up to 10 m high with 3–5 m long leaves/petiole [27]. The entire raffia palm tree is usable. The raffia nut is commonly used to extract oils for cosmetics. A kind of wine is produced from raffia sap, and its fibers are used for carpets, ropes, and handicrafts [28]. As a major producer of natural fibers and taking a unique position among South American countries, only recently in Brazil [31] have the raffia fibers from the Amazon region, also known as Jupati fiber, started to be considered as addition to polymer composites. The first work reporting on the potential of raffia fibers predicting a possible use in composites was that of Elenga et al. [29] in 2009. They described the physical and mechanical properties of the *Raphia textilis* fiber and suggested that the atypical alveoli structure (honeycomb-like and scales) of this species could help in the interface adhesion with a composite matrix. Rodrigue et al. [30] also mentioned the possibility of using the *Raphia vinifera* in composites. However, in their paper, only the variation of mechanical properties along the stem of this raffia fiber was investigated. In fact, none of these studies have actually evaluated raffia composites; these works focused exclusively on the characterization of the fiber. The work of Obasi et al. [32] in 2013 was the first to report on the use of the raffia fibers as a possible reinforcement by stiffening the composites. The properties of *Raphia farinifera* fiber randomly dispersed into high-density polyethylene (HDPE) composite, with different fiber loadings of 0 to 60 wt% were analyzed. The addition of 60 wt% of raffia fiber into the composite resulted in a Young’s modulus 2.5 times greater than that of neat HDPE. However, no statistical validation was presented to support this stiffening. By contrast, a decrease in both elongation and tensile strength was observed. Moreover, the higher loading content of fiber resulted in higher water absorption, which was reduced by around 30% with the addition of the maleic anhydride-graft-polyethylene (MA-g-PE) in their composites [32]. The reduction in tensile strength of raffia composites was also observed by Rodrigues et al. [33], who investigated the influence of the pressure level in the mechanical properties of raffia composites produced by the vacuum infusion process. The composites with 45 vol% of aligned raffia fibers disclosed a tensile strength of around 24 MPa, which was 30% lower than that of plain polyester (~34 MPa), although no significant difference was verified for both vacuum pressure levels. Table 1 presents the reported tensile strength and Young’s moduli of these earlier works [32,33] on raffia fiber composites. In this table the main point of discussion is if there exists a reinforcement effect promoted by the incorporation of raffia fiber into the polymer matrix. The single point results of Obasi et al. [32] did not allow determining a statistical interval of precision. Consequently, their values in Table 1 lack standard deviation, which does not guarantee the apparent increase shown in Young’s moduli. In regard to the work of Rodrigues et al. [33], results in both raffia aligned fiber and fabric, although showing statistical precision, failed to indicate Young’s modulus of the polyester matrix. The absence of this reference does not permit claiming a stiffening effect caused by raffia fiber/fabric addition to the polyester matrix.

As for other properties, Foadieng et al. [34] evaluated the thermal properties of raffia bamboo, which is the stem of the palm tree. They reported thermal conductivity of 0.07 W/m·K, smaller than some timbers [35], which makes the raffia bamboo a good insulation material that could be used in the structures such as houses, drying-lofts, and ceilings [34]. A hybrid sandwich composite based on raffia and glass fibers was produced and the effect of alkaline treatment of raffia fibers on the structural, thermal, and mechanical properties were reported by Ouarhim et al. [36]. The results showed higher thermal and mechanical properties for raffia-treated fiber composite in comparison to untreated raffia fiber-based sandwich composite. A different approach was taken by Overah et al. [37], who produced nanocomposites of magnetite and *Raphia farinifera* to use as an absorber of heavy metal ions. They found a greater absorption of heavy metal ions, especially Pb^2+^, for the nanocomposites with higher fiber content (magnetite/raffia ratio of 1:3), making it a good choice to be applied in contaminated wastewater.

Although several studies [1,2,3,4,5,6,7,8,9,10,11,12] have been shown the potential of NLF application in composites, natural fibers are not a challenge-free alternative. The heterogeneity and hydrophilicity are some shortcomings of NLFs [7,24]. One way to improve the performance of natural fiber-based composites is the treatment by the chemical modification of the NLF surface [38]. In order to improve the interfacial adhesion between natural fibers and the polymeric matrix, several chemical treatments have been extensively investigated, such as alkali, silane, benzoyl, acetylation, acrylation, permanganate, graphene-based coating, and stearic acid [39,40,41,42]. The alkali treatment is the most commonly used to partially remove the lignin, hemicellulose, wax, and oils covering the external surface of natural fibers, enhancing the matrix–fiber interface and, consequently, the composites mechanical properties [10]. This effect was observed by Mazzanti et al. [43] for hemp-PLA composites, in which an increase of ~16% in Young’s modulus was exhibited by the addition of 6 wt% alkali-treated hemp fiber in comparison to the untreated fiber composite. The authors reported that the alkali treatment promotes the bundle opening and individualization of thin fibers, helping in their distribution into the matrix, which may have a beneficial effect on the mechanical properties [43]. Hence, in this work, polyester composites with raffia fibers (*Raphia vinifera*), randomly dispersed, were produced and the effect of both the fiber alkali treatment and fiber length on the mechanical properties were for the first time evaluated. Moreover, based on the only previous tensile results of raffia polymer composites performed so far [32,33], which still cast doubts on a possible reinforcement effect, the present work conducted a statistical analysis by ANOVA and the Tukey test to elucidate this question.

## 2. Materials and Methods

### 2.1. Materials

The unsaturated terephthalate-based polyester resin (Arazyn AZ 1.0 #34) and the catalyst methyl-ethyl-ketone peroxide (MEK), PERMEC D-45, both supplied by Ara Química SA (São Paulo, Brazil) were used as the polymer matrix. Two ratios, 0.7 and 1 vol%, of the hardener catalyst, were evaluated for the curing of the polyester resin. The raffia fibers (*Raphia vinifera)* used in this investigation were obtained from the local market of Belém, PA, in the north region of Brazil. These fibers, shown in the insert of Figure 2, were first manually cut in three different lengths, 5, 10, and 15 mm, and then alkali-treated to remove noncellulosic impurities from the fiber surface. The alkali treatment was conducted in a 10 wt% sodium hydroxide (NaOH) solution, under ultrasonic stirring at room temperature for 1 h. During the treatment, the ratio of fiber/solution was kept between 0.075 and 1 g/mL. After that, the fibers were washed and dried at room temperature (25.8 °C) for 48 h.

### 2.2. Processing of Composites

**Figure 3:**
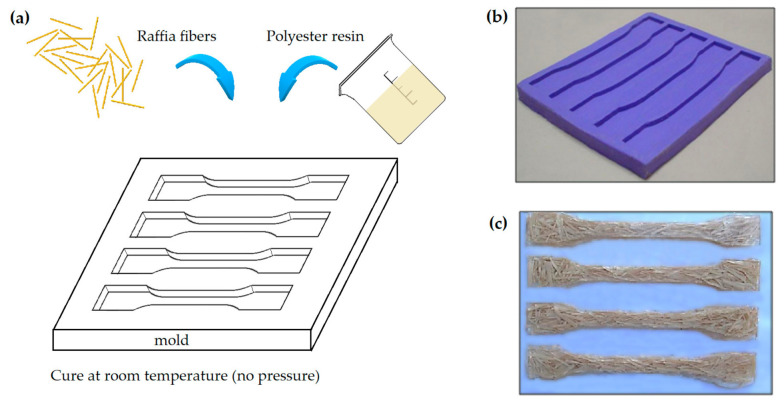
Design and manufacturing of raffia fiber composite: (**a**) process scheme; (**b**) mold; and (**c**) specimens.

Composites were produced with randomly dispersed fibers by a hand lay-up process, in a silicone mold, schematically shown in Figure 3. For this, the mass fraction of fiber was defined by the maximum volumetric capacity of the mold to accommodate the reinforcement without pressure, resulting in ~10 wt%. Eight polyester composite samples, in both untreated and treated condition, were produced for each fiber length. In addition, pure polyester samples, with different ratios of hardener catalyst, were prepared as control groups. Overall, a total of 64 samples of 10 wt% raffia fiber reinforced unsaturated polyester composites with fiber alkali-treated or untreated and different fiber lengths (5, 10 or 15 mm) as well as polymer cured with either 1.0 or 0.7 vol% of catalyst hardener were investigated.

### 2.3. Tensile Tests

The mechanical properties of the composites, in the different aforementioned conditions, were determined by tensile tests. These tests were conducted according to the ASTM D638 standard [14] using an AROTEC universal testing machine (São Paulo, Brazil), with a 5 kN load cell at a crosshead speed of 5 mm/min. Type I dimensions as per standard [14] were used to produce the specimens. The maximum value attained in the digitally recorded stress–strain curve of each tensile test was considered as the material tensile strength while the stress/strain ratio (maximum value) up to the yield point was computed as the Young’s moduli. No outside physically attached extensometer was used to measure strains. Only digitally interpreted crosshead speed and specimen gage length were used to calculate the strain through the machine electronic interface program.

### 2.4. Statistical Analysis

Analysis of variance (ANOVA) was applied using the F test to verify whether there was a significant difference between the results obtained for tensile strength and Young’s moduli. The 95% confidence level was adopted and the Tukey test complemented this statistical analysis to quantitatively assess the most prominent value by means of the lower significant difference.

### 2.5. Additional Characterization

The raffia fiber dimensions and their frequency distributions were determined by an optical microscope, model BX53M, Olympus (Tokyo, Japan). Forty fibers, in both untreated and alkali-treated conditions, were randomly selected for statistical analysis of their dimensions, which were measured at five equally spaced positions along the fiber length, as described elsewhere [45]. In order to study the failure mechanisms of each fabricated composition, the fracture surfaces of the specimens were also analyzed after the mechanical tests. In addition, FTIR analysis was carried out to verify the chemical interaction between raffia fiber and polyester matrix in a Thermo Fisher Scientific equipment, model Nicolet iS50 (Waltham, MA, USA), using a mid-infrared range (4000–400 cm^−1^). The morphology of the fracture surface was performed by scanning electron microscopy (SEM) in a model VEJA 3 SBU, TESCAN (Brno, Czech Republic), using secondary electrons at 20 kV accelerated voltage. All samples were gold sputtered before being subjected to SEM investigation.

## 3. Results and Discussion

### 3.1. Frequency Distribution of Raffia Fiber Dimensions

**Figure 4:**
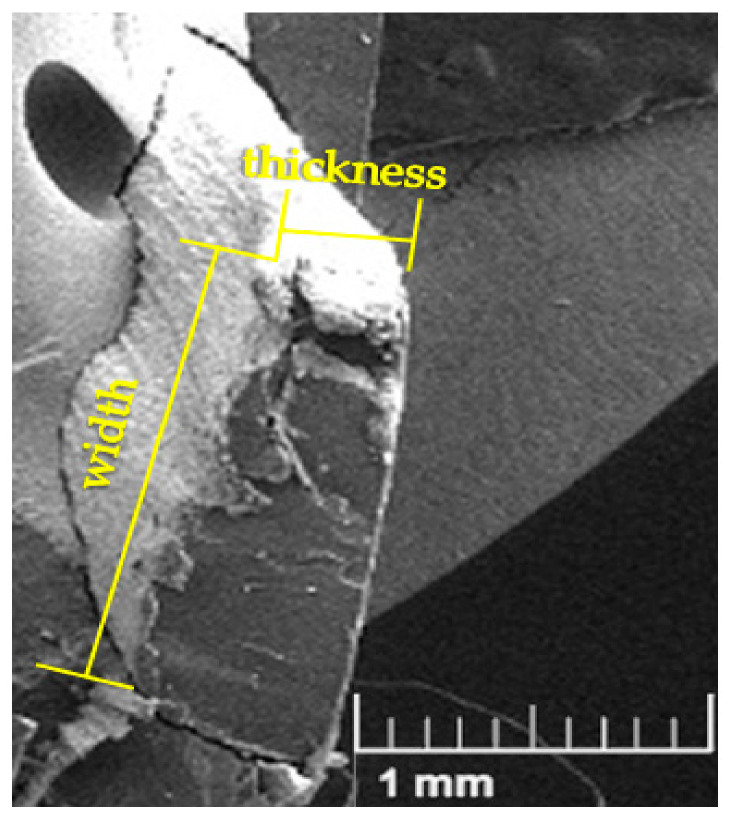
Scanning electron microscopy (SEM) image of an untreated raffia fiber cross-section.

**Figure 5:**
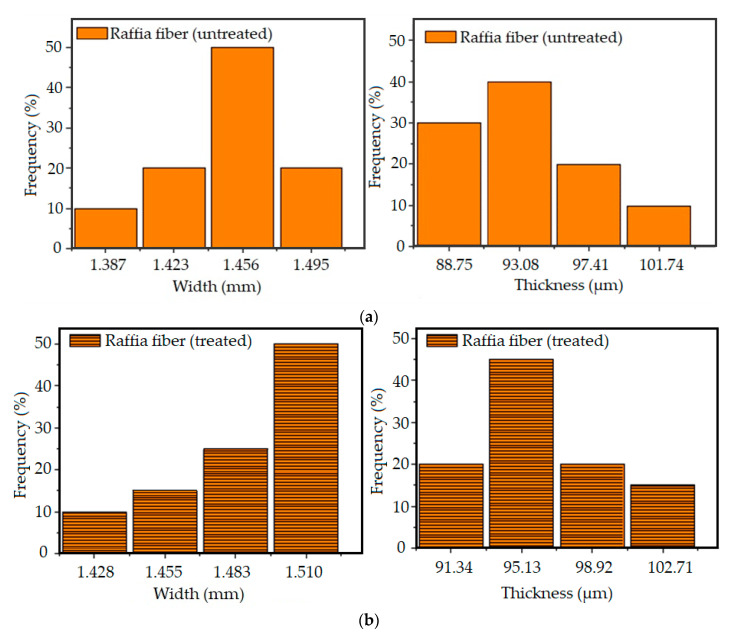
Frequency distribution of the widths and thicknesses of raffia fibers: (**a**) untreated; and (**b**) alkali-treated.

The raffia fiber Figure 4 exhibits an almost rectangular cross-section. The fiber length, corresponding to the major dimension, was cut in sizes of 5, 10, and 15 mm as an investigated variable. The rectangular fiber cross-section has dimensions with clearly different sizes. The greater is indicated as the “width” while the shorter as the “thickness” in Figure 4. Figure 5 presents the resulting histograms for the distribution of thickness and widths for conditions, untreated (Figure 5a) and alkali-treated raffia fibers (Figure 5b). The untreated fibers exhibited average widths and thicknesses of 1.450 ± 0.032 mm and 93.513 ± 4.191 μm, respectively. After treatment, these fibers showed a dimensional increase of 2% in width (1.487 ± 0.028 mm), and 3.5% in thickness (96.267 ± 3.709 μm), which could be attributed to the volumetric expansion that the fiber suffered during the chemical treatment.

### 3.2. Tensile Test of Neat Polyester (Matrix)

**Figure 6:**
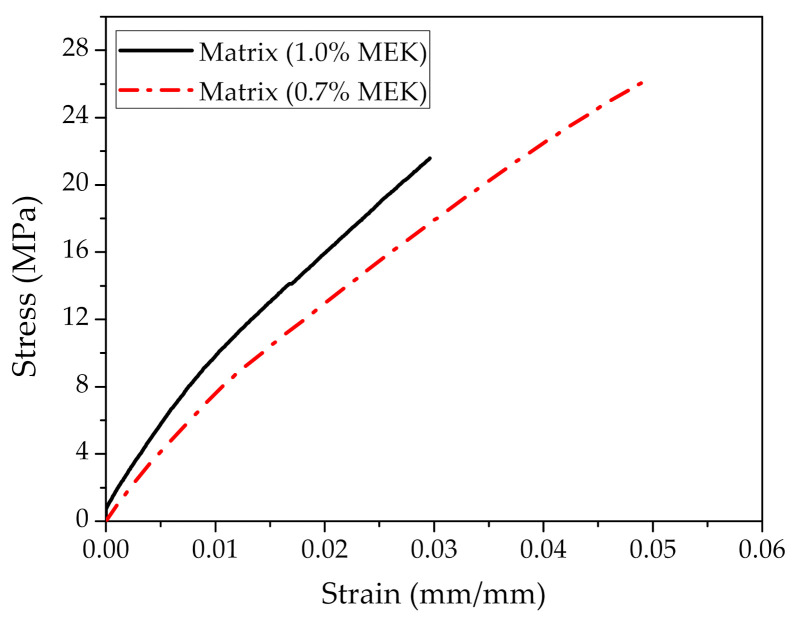
Comparative tensile test curves of neat polyester samples with different volume fractions (0.7 and 1 vol%) of the methyl-ethyl-ketone peroxide (MEK) catalyst.

Figure 6 shows the effect of different ratios of hardener catalyst on tensile test results of the neat polyester samples. It can be noted that there is a higher stiffness for polyester with 1.0 vol% of MEK catalyst (~0.86 GPa), in comparison to the one with 0.7 vol% (~0.80 GPa). Moreover, the values of deformation were higher in the samples with 0.7 vol% of MEK. It is also worth mentioning that both polyester curves in Figure 6 exhibit a relatively ductile behavior under tensile test, but undergo a sharp fracture with no energy absorption capacity after matrix rupture. Due to the high stiffness of polyester with 1.0 vol% of MEK, only composites with this matrix had their tensile properties evaluated. Moreover, 1.0 vol% MEK was the recommended ratio by the catalyst maker.

### 3.3. Raffia Fiber Reinforced Polyester Composites

**Figure 7:**
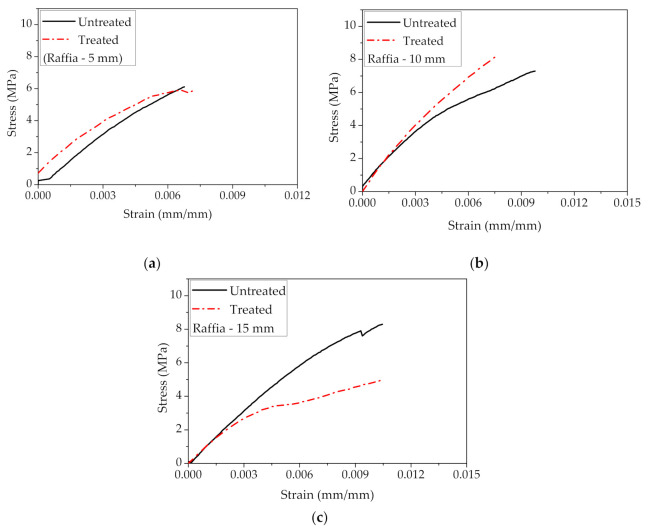
Stress-strain curves of raffia fiber composites under alkali treated and untreated conditions with different fiber lengths: (**a**) 5 mm; (**b**) 10 mm; and (**c**) 15 mm.

Figure 7 illustrates the typical stress–strain curves of raffia fiber composites under alkali-treated and untreated conditions for the different fiber lengths. It can be noted that the highest tensile strength and deformation are obtained by raffia composites (untreated) with a length of 15 mm (Figure 7c), presenting 8.50 MPa and 1.79%, respectively. This behavior was also observed by Fadele et al. [46] for the tensile strength of their alkali-treated *Raphia farinifera* fibers. After chemical treatment with a 10 wt% NaOH solution, the fiber strength was reduced by 47%, and its deformation was slightly changed [46]. Although the authors verified an increase of 22% in cellulose content and a decrease of ~8% in the lignin content, they attributed the decreasing of the fiber tensile strength to the presence of voids/flaws, which are sources of stress concentration [46]. In addition, it is possible that the treatment parameters, such as concentration, immersion time, and temperature, were not adequate. Several studies on the degradation of the tensile strength of natural fibers after chemical treatment reported on the influence of these parameters [46,47,48,49]. For example, Mahjoub et al. [47] verified a decrease in the tensile strength of kenaf fibers for higher values of NaOH solution concentration and immersion time. Similar results were observed for flax and abaca fiber, presenting a lower tensile strength by increasing immersion time in 3% NaOH solution [49].

Table 2 presents the tensile test results of this work. As aforementioned, to our knowledge, there are just two works which reported on raffia-based composites [32,33]. Obasi et al. [32] discussed the effect of the fiber mass fraction and MA-g-PE as a compatibilizer on the tensile properties of HDPE composite. Their results, in Table 1, display that the MA-g-PE barely changes the mechanical properties of the HDPE composites. By contrast, the fiber content strongly influences the tensile strength, reducing it by almost 70% for the composite with 60 wt% of raffia fiber in comparison to the neat HDPE. Even so, they verified improvement in the biodegradability of HDPE by adding raffia fiber [32]. Similar behavior on tensile strength was observed in the present study, not caused by fiber content, but by the fiber length, which exhibited a higher decrease for composites with the shortest fibers (Table 2).

**Figure 8:**
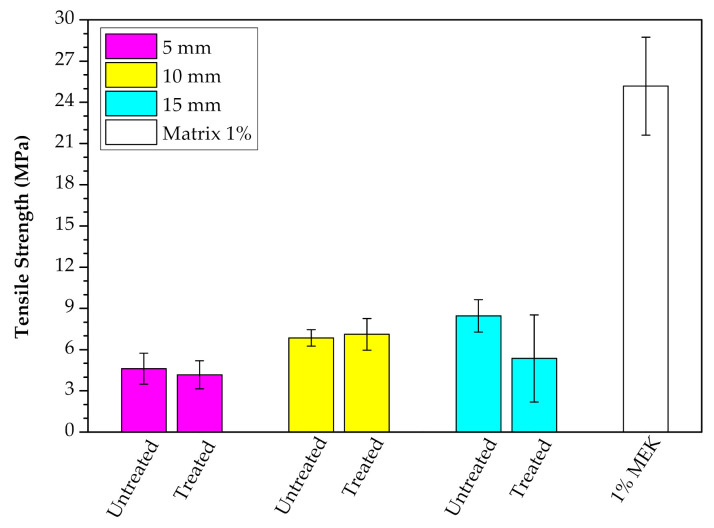
Tensile strength of raffia fiber composites under alkali treated and untreated conditions, with 5, 10, and 15 mm of fiber length.

**Figure 9:**
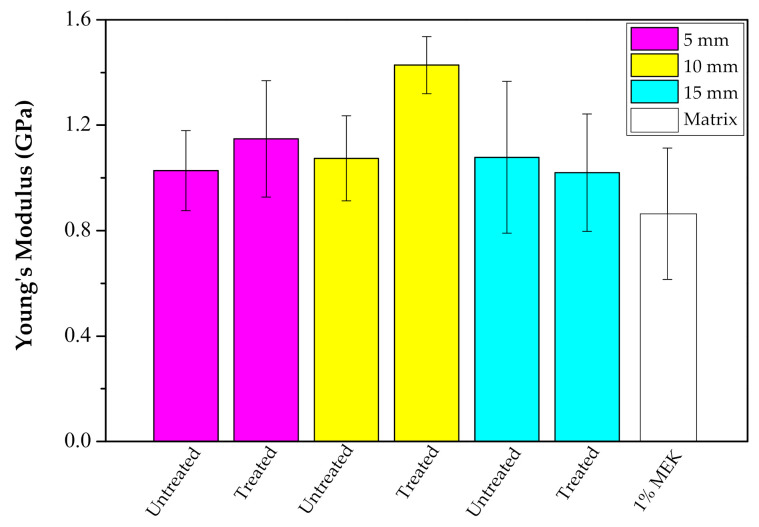
Young’s modulus of raffia fiber composites under alkali treated and untreated conditions, with 5, 10, and 15 mm of fiber length.

Although the raffia fiber has relatively high cellulose (53 wt%) and lignin (24 wt%) contents [28], which are responsible for its strength, Figure 8 indicates that this fiber acted only as filler into the polyester matrix. This may have occurred due to either an unsuitable processing of the composite or a weak interfacial fiber/matrix adhesion. As further shown, porosity and lack of fiber adhesion to the matrix are revealed by SEM fractographs. The relatively small amount (10 wt%) of added fiber is not expected to bring difficulty in the composite processing. Therefore, we believe that poor raffia fiber adhesion to the polyester should be the main reason for all composites relatively low tensile strength, as compared to the plain matrix in Figure 8. It should also be mentioned that the atypical rectangular cross-section of the raffia fiber, shown in Figure 4, might introduce internal stress concentration in the fiber/matrix interface due to the rectangular sharp corners. Indeed, the other works on raffia fiber composites using compression molding [32] and vacuum infusion [33] reported similar low tensile strength in comparison to their matrices (Table 1) although having strength superior to our values in Figure 8. However, the composite with treated raffia fiber with a length of 10 mm, exhibits the highest Young’s modulus, exceeding by 66% the stiffness of the neat polyester, shown in Figure 9, which reveals a reinforcement effect. This significant difference was confirmed through ANOVA and Tukey statistical analyses shown in Table 3. Based on this table, is possible to claim, with a 95% confidence level, that the raffia alkali-treated fiber (10 mm) composite was the best condition since the *p*-value is lower than 5% and the difference between this composite and neat polyester is higher than the truly significant difference (HSD). It can be noted that the length had an effect regarding the tensile strength as shown in ANOVA and Tukey results of Table 4. The composite with the highest raffia fiber length, 15 mm, in untreated condition exhibits an increase of over 2 times in tensile strength in comparison to the composite with a 5 mm (alkali-treated) condition in Figure 8.

**Figure 10:**
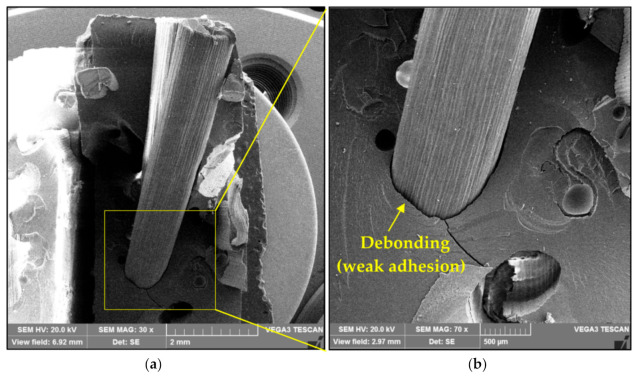
SEM images of fracture surfaces of composites with untreated raffia fibers: (**a**) with a length of 15 mm, and (**b**) detail of the fiber debonding.

**Figure 11:**
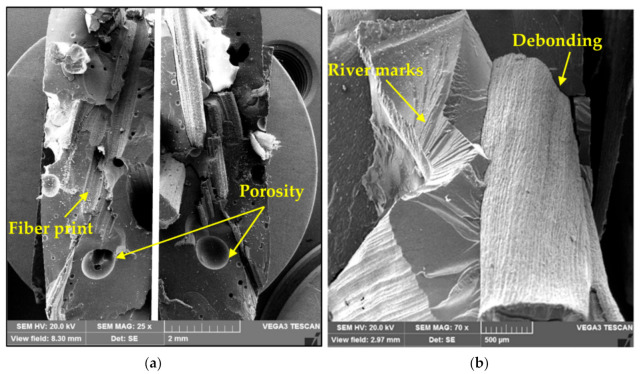
SEM images of fracture surfaces of composites with alkali-treated raffia fibers with a length of 15 mm: (**a**) defects in both parts of the sample, and (**b**) fracture mechanisms.

SEM images of fracture surfaces of composites with untreated and alkali-treated raffia fibers are shown in Figure 10 and Figure 11, respectively. The alkali treatment removes the non-crystalline constituents inherent to natural fibers such as hemicellulose and waxes, which did not contribute to a stronger bonding between the raffia fiber and polyester matrix. Consequently, the alkali treatment did not improve the transfer of loads from the matrix to the fiber. Hence, for both cases, it can be observed the predominance of failure mechanism associated with weak interfacial adhesion and fiber pullout, which were also verified by Rodrigues et al. [33]. These are indicated by the fiber debonding and fiber print in the matrix. Moreover, no polyester matrix attached to the raffia fiber is shown in Figure 10 and Figure 11. The presence of porosity and river marks, characteristic of the brittle matrix fracture, are also noted in Figure 11.

**Figure 12:**
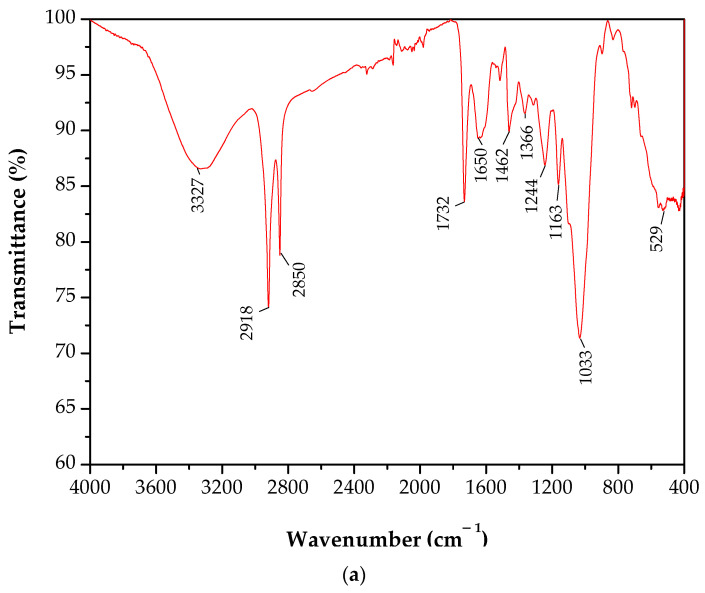

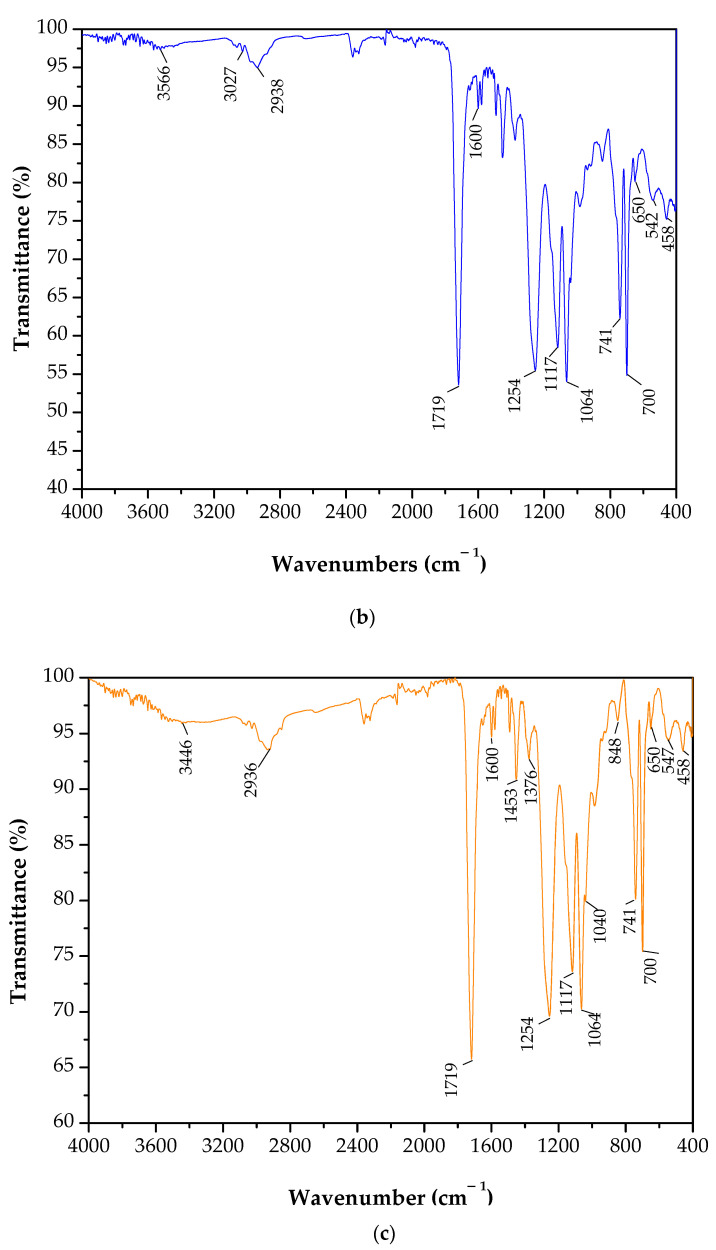
FTIR spectra of raffia fiber and its composites: (**a**) raffia fiber; (**b**) polyester; and (**c**) raffia composite.

Figure 12 shows the FTIR spectra of raffia fiber and its composites. The wavenumbers and assignments of FTIR bands are summarized in Table 5. It can be observed that the raffia fiber consists of alkene, esters, aromatics, ketone, and alcohol, with different oxygen-containing functional groups, such as OH (3327 cm^−1^), C=O (1732 cm^−1^), O–CH_3_ (1462 cm^−1^), C–O–C (1244 cm^−1^), and C–O (1033 cm^−1^). The band in the range of 3600 to 3000 cm^−1^ is related to the hydroxyl groups (O–H) stretching of the hydrogen bond in the cellulose and hemicellulose [50]. The methyl group vibration, which is a typical molecular structure of the natural fiber, appears at 2918 and 2850 cm^−^¹, given by the elongation of the aliphatic C–H bonds [50,51]. In addition, it is noticed that these characteristic vibrations are present in lignin. The band at 1732 cm^−1^ is attributed to the carbonyl groups (C=O) while the stretching of the C–O–C group (1244 cm^−1^) is associated with the vibration in cellulose glycemic rings [52]. Concerning the raffia fiber/polyester composites, the O–H stretching is observed in the range of 3600 to 3400 cm^−1^, and stretching vibrations of C–H is verified in the ranges from 3100 to 2900 cm^−1^ and from 1460 to 1250 cm^−1^. This latter one corresponds to the CH_2_ and CH_3_ groups’ vibrations. It can be noted the vibrations of C–H groups have approximately the same transmittance bands for both composites. The stretching vibration of the C=O group occurs at 1728 cm^−1^. The three bands at 1600, 1580, and 1492 cm^−1^ are assigned to aromatic ring stretching and appear at the same positions for the composites, which indicates that no change occurred in chemical interaction between the aromatic ring and the raffia fiber. According to Cecen et al. [53], the vibrations at 1453 and ~1380 cm^−1^ correspond to asymmetric and symmetric bending of methyl groups, respectively. In addition, the band at 1254 cm^−1^ may be attributed to the CH_2_ twist vibration and C–O stretching vibrations which occurs at 1117 cm^−1^. However, no expressive change was observed in the FTIR spectrum of raffia composite, which corroborates the aforementioned results of mechanical properties and SEM analyses.

## 4. Conclusions

- The addition of 1.0 vol% of MEK catalyst into neat polyester resulted in the highest Young’s modulus ~0.86 GPa, which was ~10% higher than polyester with 0.7 vol% of MEK.
- Tensile strength results indicated that the raffia fiber acted only as a filler into the polyester composites, which may be associated with either an unsuitable processing of the composite or weak interfacial fiber/matrix adhesion. In spite of that, an increase in the Young’s moduli of the composites was obtained in comparison with that of the polyester matrix.
- Statistical analyses by ANOVA and the Tukey test confirmed for the first time a stiffening effect caused by 10 wt% raffia fibers with 10 mm in length to the unsaturated polyester matrix composite.
- The tensile results also disclosed the effect of fiber length on the mechanical strength of the composites. The highest tensile strength was reached by the composite with a higher length (15 mm) raffia fiber, in untreated condition, which represented an increase of more than 100% in comparison to the composite with 5 mm (alkali-treated) fiber. All composites with alkali-treated raffia fiber presented similar tensile strength values that, according to ANOVA, are lower than those for the untreated condition.
- SEM analyses revealed the predominance of a failure mechanism associated with weak interfacial adhesion and porosity, even for the composites with alkali-treated raffia fibers.
- FTIR analysis failed to disclose any significant change in the raffia composite transmittance bands, which corroborated the relatively unaltered mechanical properties and weak interfacial fiber/polyester adhesion.
- To date, based on the Scopus metrics, there are very few studies on raffia fiber composites. In addition to confirming a stiffening, the effects of raffia fiber length and treatment in mechanical properties were also disclosed. Hence, this study provides information filling the current knowledge gap of raffia fiber, which aims to valorize this abundant and unexploited Brazilian resource.

## Figures and Tables

**Table 1:** Earlier results of tensile tests of Raffia fiber composites [32,33].

Composite	Manufacturing Process	Condition	Fiber Content	Fiber Length (mm)	Tensile Strength (MPa)	Young’s Modulus	Ref.
						(GPa)	
Neat HDPE Matrix	Compression molding at 150 °C	Untreated	0 wt%	NA *	30	0.31	[32]
HDPE/raffia fiber (powder)			15 wt%		18	0.33	
			30 wt%		15	0.42	
			45 wt%		13	0.60	
			60 wt%		10	0.79	
Neat HDPE Matrix		Treated with MA-g-PE	0 wt%		29	0.31	
HDPE/raffia fiber (powder)			15 wt%		22	0.33	
			30 wt%		18	0.45	
			45 wt%		15	0.62	
			60 wt%		12	0.81	
Neat Polyester Matrix	Vacuum infusion process	53.3 kPa	0 wt%	NA *	34.36	NI **	[33]
Polyester/raffia fiber (aligned)		53.3 kPa	45 vol%		23.59 ± 5.52	2.325 ± 0.180	
		101.3 kPa	40 vol%		22.97 ± 1.58	1.303 ± 0.090	
Polyester/raffia fabric		53.3 kPa	35 vol%		14.42 ± 0.90	1.010 ± 0.059	
		101.3 kPa	43 vol%		20.27 ± 1.88	0.913 ± 0.124	

* NA: not applicable. ** NI: not indicated.

**Table 2:** Results of tensile tests of samples of Raffia fiber polyester composites.

Composite	Manufacturing Condition		Fiber Content	Fiber Length (mm)	Tensile Strength (MPa)	Young’s Modulus	Ref.
						(GPa)	
Neat Polyester Matrix	Hand lay-up process	1% MEK	0 wt%	NA *	25.18 ± 3.56	0.86 ± 0.25	PW **
Polyester/raffia fiber (randomly dispersed)		Untreated	~10 wt%	5	4.61 ± 1.12	1.03 ± 0.15	
		Treated		5	4.17 ± 1.02	1.15 ± 0.22	
		Untreated		10	6.85 ± 0.59	1.07 ± 0.16	
		Treated		10	7.12 ± 1.16	1.43 ± 0.11	
		Untreated		15	8.46 ± 1.18	1.08 ± 0.29	
		Treated		15	5.36 ± 3.17	1.02 ± 0.23	

* NA: not applicable. ** PW: present work.

**Table 3:** Analysis of variance (ANOVA) and Tukey results of Young’s moduli of neat polyester and raffia composites.

**ANOVA**	**Source**	**Sum of Squares**	**Degrees of Freedom**	**Mean of Squares**	**F (Calculated)**	**F Critical**	***p*-Value**
	Treatment	0.889	6	0.148	3.405	2.445	0.012
	Residual	1.218	28	0.0435			
	Total	2.107	34				
**Tukey Test**	**Degrees of Freedom (Total)**		**q (Tabled)**	**Mean of Squares (Residual)**			**HSD**
	28		4.49	0.04			0.42

**Table 4:** ANOVA and Tukey results of tensile strength of neat polyester and raffia composites.

**ANOVA**	**Source**	**Sum of Squares**	**Degrees of Freedom**	**Mean of Squares**	**F (Calculated)**	**F Critical**	***p*-Value**
	Treatment	1628.936	6	271.489	67.561	2.445	2.34 × 10^−15^
	Residual	112.515	28	4.018			
	Total	1741.451	34				
**Tukey Test**	**Degrees of Freedom (Total)**		**q (Tabled)**	**Mean of Squares (Residual)**			**HSD**
	28		4.49	4.018407			4.025208

**Table 5:** Assignment of the main FTIR bands of raffia fiber and its polyester composites.

Material	Wavenumber (cm^−1^)	Assignment
Raffia fiber	3600–3000	OH stretching
	2950–2840	C–H_n_ stretching
	1732	C=O stretching
	1510–1560	
	1650–1630	C=C (Benzene stretching ring)
	1462	O–CH_3_
	1440–1400	OH bending
	1402	CH bending
	1244	C–O–C stretching
	1033	C–O stretching and C–O deformation
	700–400	C–C stretching
Raffia/polyester composite	3600–3400	O–H stretch
	3060	Aliphatic C–H stretch
	3026	
	~2936	
	1719	C=O stretch
	1600	Aromatic ring stretch
	1580	
	1492	
	1453	CH_3_ asymmetrical bend
	1376	CH_3_ symmetrical bend
	1254	CH_2_ twist
	1117	C–O stretch
